# Human Islet Amyloid Polypeptide Overexpression in INS-1E Cells Influences Amylin Oligomerization under ER Stress and Oxidative Stress

**DOI:** 10.3390/ijms222111341

**Published:** 2021-10-20

**Authors:** Yeong-Min Yoo, Seong Soo Joo

**Affiliations:** 1 East Coast Life Sciences Institute, College of Life Science, Gangneung-Wonju National University, Gangneung 25457, Gangwon-do, Korea; 2 Department of Marine Life Sciences, College of Life Science, Gangneung-Wonju National University, Gangneung 25457, Gangwon-do, Korea

**Keywords:** human amylin or islet amyloid polypeptide, overexpression, ER stress, oxidative stress, INS-1E cells

## Abstract

Human amylin or islet amyloid polypeptide (hIAPP) is synthesized in the pancreatic β-cells and has been shown to contribute to the pathogenesis of type 2 diabetes (T2D) in vitro and in vivo. This study compared amylin oligomerization/expression and signal transduction under endoplasmic reticulum (ER) stress and oxidative stress. pCMV-hIAPP-overexpressing INS-1E cells presented different patterns of amylin oligomerization/expression under ER stress and oxidative stress. Amylin oligomerization/expression under ER stress showed three amylin oligomers of less than 15 kDa size in pCMV-hIAPP-overexpressing cells, while one band was detected under oxidative stress. Under ER stress conditions, HIF1α, p-ERK, CHOP, Cu/Zn-SOD, and Bax were significantly increased in pCMV-hIAPP-overexpressing cells compared to the pCMV-Entry-expressing cells (control), whereas p-Akt, p-mTOR, Mn-SOD, catalase, and Bcl-2 were significantly decreased. Under oxidative stress conditions, HIF1α, p-ERK, CHOP, Mn-SOD, catalase, and Bcl-2 were significantly reduced in pCMV-hIAPP-overexpressing cells compared to the control, whereas p-mTOR, Cu/Zn-SOD, and Bax were significantly increased. In mitochondrial oxidative phosphorylation (OXPHOS), the mitochondrial complex I and complex IV were significantly decreased under ER stress conditions and significantly increased under oxidative stress conditions in pCMV-hIAPP-overexpressing cells compared to the control. The present study results demonstrate that amylin undergoes oligomerization under ER stress in pCMV-hIAPP-overexpressing cells. In addition, human amylin overexpression under ER stress in the pancreatic β cells may enhance amylin protein aggregation, resulting in β-cell dysfunction.

## 1. Introduction

Although human amylin or islet amyloid polypeptide (hIAPP) and rat IAPP (rIAPP) have 83% sequence identity, hIAPP rapidly aggregates into amyloid fibrils in vitro while rIAPP does not form fibrils under the same experimental conditions [1,2,3]. When comparing these two peptides sequences, the variation is evident in the region spanning residues 20–29. Westermark et al. evaluated the aggregation ability of peptides containing residues 20–29 and confirmed that they could form amyloid fibrils in vitro [4]. Thus, amino acid residues 20–29 of hIAPP play an essential role in the aggregation of amylin into amyloid and may act as a significant determinant of amyloid growth [2,5]. Previous studies have shown that hIAPP aggregation is initiated by an intrinsically disordered 37 amino acid peptide and the chemical properties of amino acids, including charge, hydrophobicity, and aromaticity, influence hIAPP aggregation [2,6]. Therefore, the molecular determinant in the production of hIAPP amyloid deposits is the amino acid composition of the peptide. For instance, some studies have suggested that N-terminal domain variants are less toxic to INS-1 cells but are involved in the modulation of toxicity and are in the critical position [2,7,8,9]. In particular, in a recent study, Nguyen et al. [10] reported that adding a single methylene group by substituting Gln for Asn at position 21 (N21Q) of IAPP resulted in the formation of fibrils with potent cytotoxicity to INS-1E cells. They suggested that N21Q fibrillar structure enables conformational self-assembly, and the resulting IAPP assembly can be cytotoxic.

Lorenzo et al. [11] demonstrated that hIAPP is only toxic to beta cells of the adult pancreas in rats and humans. Further, the result of their study proved that selective cytotoxicity is induced by the deposition of amyloid fibrils from amylin peptides and occurs by direct contact with the cell surface. Therefore, this result indicates that the presence of amylin fibrils in the pancreas can lead to islet cell dysfunction and death, leading to type 2 diabetes (T2D) [12,13]. In addition, the cytotoxicity of hIAPP in pancreatic β cells is enhanced by several cellular stress conditions, including islet inflammation, oxidative stress, mitochondrial dysfunction, DNA damage, apoptosis, endoplasmic reticulum (ER) stress, and plasma membrane disruption [14,15]. Extracellular hIAPP aggregation is involved with ER stress response in mouse beta cells [16,17]. In addition, toxic intracellular aggregates were formed in rat pancreatic beta-cells overexpressing hIAPP, which induces defective insulin and IAPP secretion by reacting to glucose [18]. Nevertheless, the role of hIAPP overexpression and ER stress induction has not been fully elucidated. 

This study compared the amylin oligomerization/expression and cell signaling pathway under ER stress and oxidative stress conditions. ER stress is induced by thapsigargin or tunicamycin treatments, while oxidative stress is caused by serum deprivation, nitric oxide, or hydrogen peroxide treatments.

## 2. Results

### 2.1. The hIAPP Oligomerization/Expression and Signal Pathways under ER Stress Conditions

**Figure 1:**
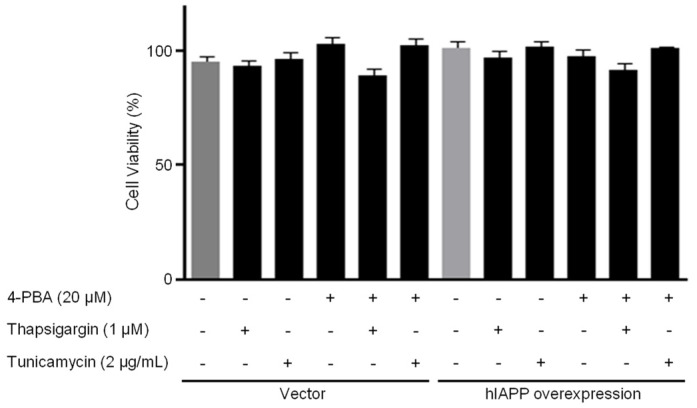
Cell viability in pCMV-Entry-expressing cells and INS1E-hIAPP-overexpressing cells under ER stress conditions. INS-1E cells were incubated in RPMI 1640 medium supplemented with 2% fetal bovine serum with/without thapsigargin (1 μM) for 6 h or tunicamycin (2 μg/mL) for 16 h and/or with/without 4-PBA (20 μM) at 37 °C with 5% CO_2_. Cell viability was analyzed by Cell Counting Kit-8. Values are presented as the mean ± SEM of three experiments.

**Figure 2:**
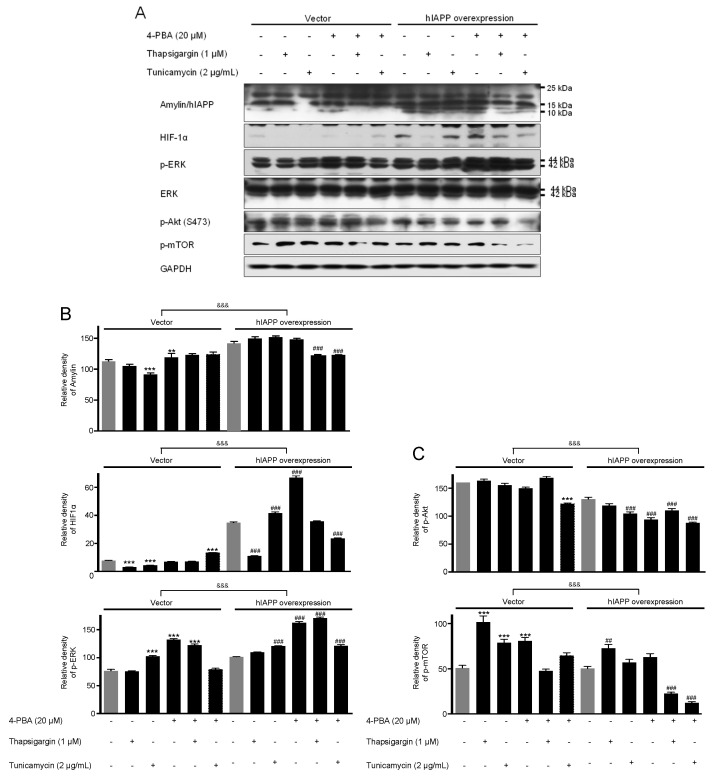
The expressions of amylin/hIAPP, HIF1α, p-ERK, p-Akt, and p-mTOR proteins in pCMV-Entry-expressing cells and INS1E-hIAPP-overexpressing INS-1E cells under ER stress conditions. INS-1E cells were incubated in RPMI 1640 medium supplemented with 2% fetal bovine serum with/without thapsigargin (1 μM) for 6 h or tunicamycin (2 μg/mL) for 16 h and/or with/without 4-PBA (20 μM) at 37 °C with 5% CO_2_. Amylin/hIAPP, HIF1α, p-ERK, p-Akt, and p-mTOR were then analyzed by Western blot (**A**). The relative amounts of Amylin/hIAPP, HIF1α, and p-ERK (**B**) and p-Akt, and p-mTOR (**C**) were quantified as described in Materials and Methods. Data represent the mean ± SEM of three experiments. ** *p* < 0.01, *** *p* < 0.001 vs. 2% FBS in pCMV-Entry-expressing cells; ^##^ *p* < 0.01, ^###^ *p* < 0.001 vs. 2% FBS in INS1E-hIAPP-overexpressing cells; ^&&&^ *p* < 0.001, pCMV-Entry-expressing cells vs. INS1E-hIAPP-overexpressing cells.

**Figure 3:**
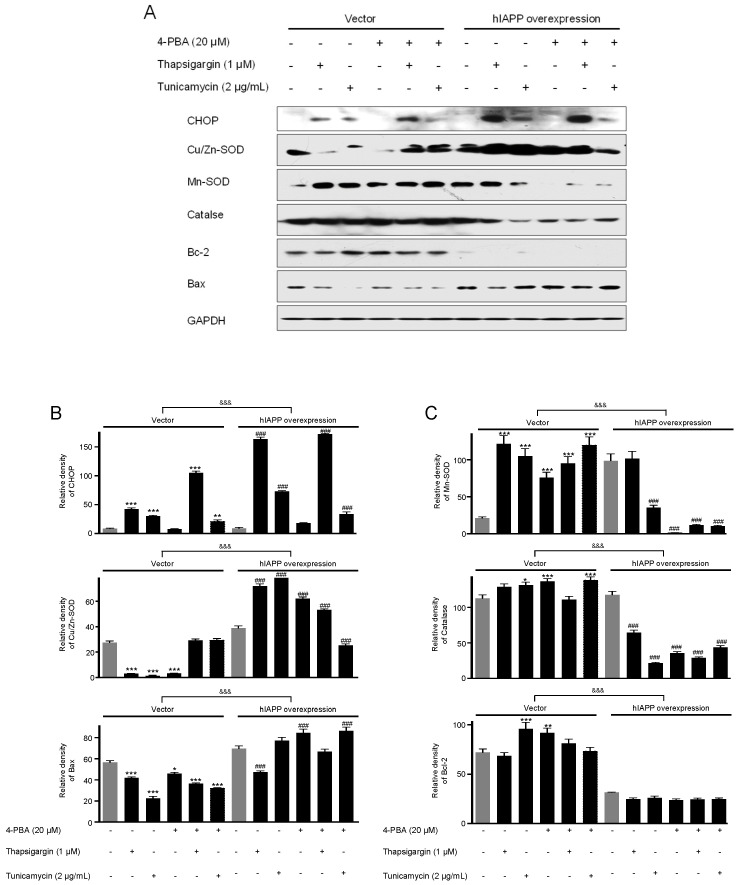
The expressions of CHOP, Cu/Zn-SOD, Mn-SOD, catalase, Bcl-2, and Bax proteins in pCMV-Entry-expressing cells and INS1E-hIAPP-overexpressing INS-1E cells under ER stress conditions. INS-1E cells were incubated in RPMI 1640 medium supplemented with 2% fetal bovine serum with/without thapsigargin (1 μM) for 6 h or tunicamycin (2 μg/mL) for 16 h and/or with/without 4-PBA (20 μM) at 37 °C with 5% CO_2_. CHOP, Cu/Zn-SOD, Mn-SOD, catalase, Bcl-2, and Bax were then analyzed by Western blot (**A**). The relative amounts of CHOP, Cu/Zn-SOD, and Bax (**B**) and Mn-SOD, catalase, and Bcl-2 (**C**) were quantified as described in Materials and Methods. Data represent the mean ± SEM of three experiments. * *p* < 0.05, ** *p* < 0.01, *** *p* < 0.001 vs. 2% FBS in pCMV-Entry-expressing cells; ^###^ *p* < 0.001 vs. 2% FBS in INS1E-hIAPP-overexpressing cells; ^&&&^ *p* < 0.001, pCMV-Entry-expressing cells vs. INS1E-hIAPP-overexpressing cells.

**Figure 4:**
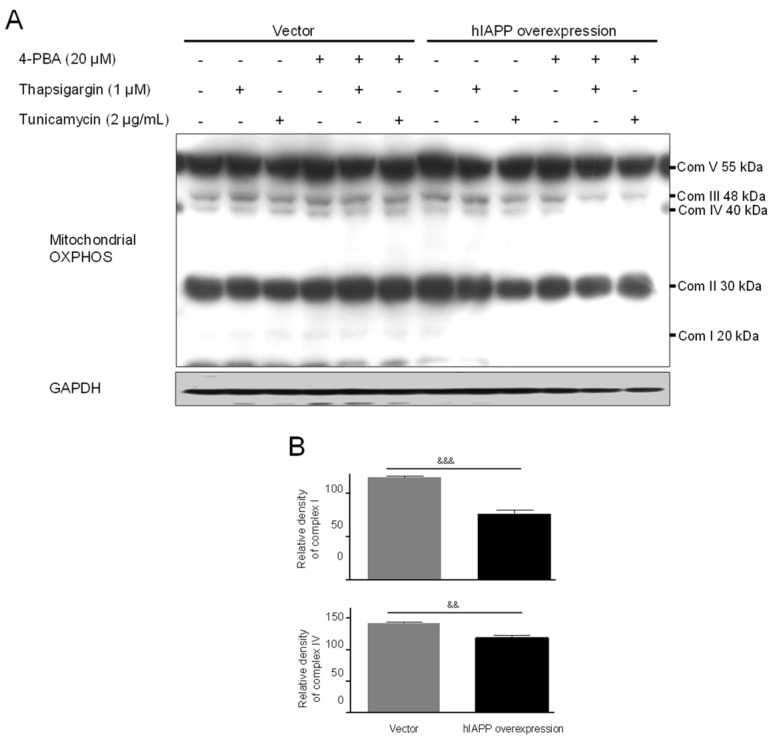
The effect of mitochondrial complex I and complex IV of OXPHOS in pCMV-Entry-expressing cells and INS1E-hIAPP-overexpressing INS-1E cells under ER stress conditions. INS-1E cells were incubated in RPMI 1640 medium supplemented with 2% fetal bovine serum with/without thapsigargin (1 μM) for 6 h or tunicamycin (2 μg/mL) for 16 h and/or with/without 4-PBA (20 μM) at 37 °C with 5% CO_2_. Mitochondrial complex I and complex IV were then analyzed by Western blot (**A**). The relative amounts of mitochondrial complex I and complex IV (**B**) were quantified as described in Materials and Methods. Data represent the mean ± SEM of three experiments. ^&&^ *p* < 0.01, ^&&&^ *p* < 0.001, pCMV-Entry-expressing cells vs. INS1E-hIAPP-overexpressing cells.

First, we investigated hIAPP oligomerization/expression and signal pathways under ER stress in INS-1E cells. ER stress is induced by thapsigargin (1 μM) or tunicamycin (2 μg/mL) treatments with/without 4-phenylbutyric acid (4-PBA, 20 μM). pCMV6-Entry and pCMV-hIAPP vectors were stably transfected in the INS-1E cells and selected with G418. The introduced hIAPP gene was confirmed by hIAPP overexpression determined using Western blot. The cell viability showed no significant change by thapsigargin or tunicamycin treatments with/without 4-PBA (Figure 1). Amylin oligomerization/expression showed three bands of amylin oligomers of less than 15 kDa size in INS-1E cells. HIF1α, p-ERK, CHOP, Cu/Zn-SOD, and Bax were significantly increased in the pCMV-hIAPP-overexpressing INS-1E cells compared to the pCMV-Entry-expressing INS-1E cells (the control), whereas p-Akt, p-mTOR, Mn-SOD, catalase, and Bcl-2 were significantly decreased (Figure 2 and Figure 3). In mitochondrial oxidative phosphorylation (OXPHOS), mitochondrial complex I and complex IV were significantly decreased under ER stress conditions in INS1E-hIAPP-overexpressing cells compared to the control (Figure 4).

### 2.2. The hIAPP Oligomerization/Expression and Signal Pathways under Oxidative Stress Conditions

**Figure 5:**
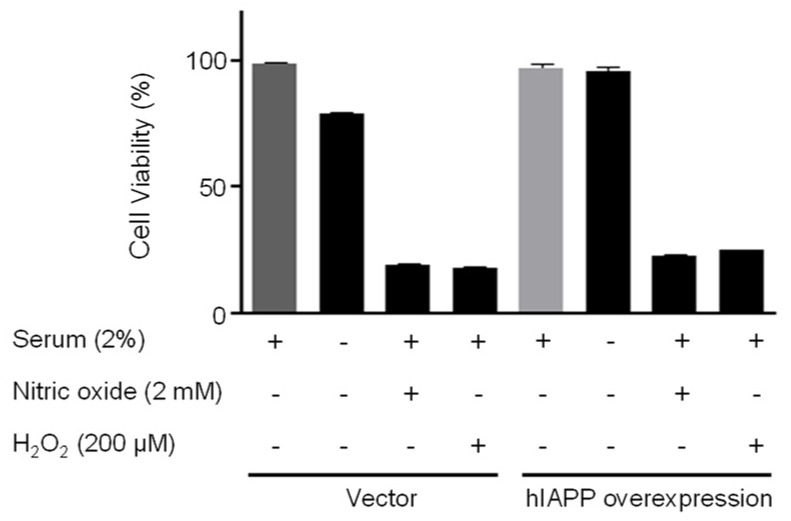
Cell viability in pCMV-Entry-expressing cells and INS1E-hIAPP-overexpressing cells under oxidative stress conditions. INS-1E cells were incubated in RPMI 1640 medium supplemented with/without 2% fetal bovine serum and/or nitric oxide (2 mM) or hydrogen peroxide (200 μM) for 24 h at 37 °C with 5% CO_2_. Cell viability was analyzed by Cell Counting Kit-8. Values are presented as the mean ± SEM of three experiments.

**Figure 6:**
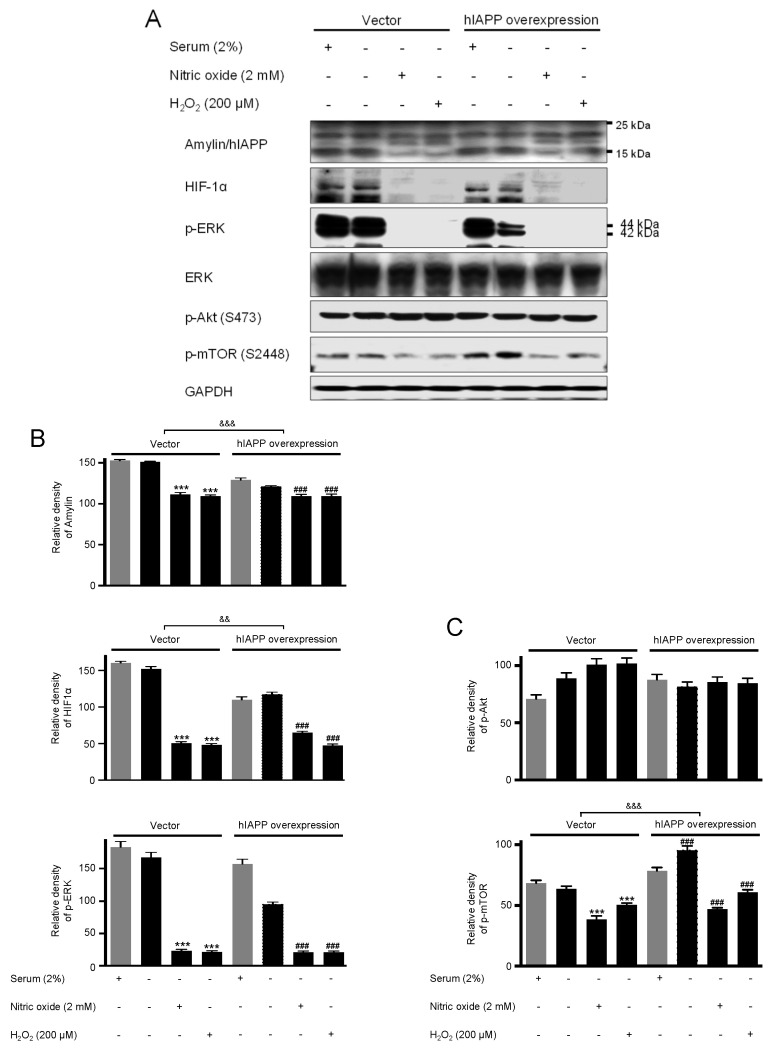
The expressions of amylin/hIAPP, HIF1α, p-ERK, p-Akt, and p-mTOR proteins in pCMV-Entry-expressing cells and INS1E-hIAPP-overexpressing INS-1E cells under oxidative stress conditions. INS-1E cells were incubated in RPMI 1640 medium supplemented with/without 2% fetal bovine serum and/or nitric oxide (2 mM) or hydrogen peroxide (200 μM) for 24 h at 37 °C with 5% CO_2_. Amylin/hIAPP, HIF1α, p-ERK, p-Akt, and p-mTOR were then analyzed by Western blot (**A**). The relative amounts of Amylin/hIAPP, HIF1α, and p-ERK (**B**) and p-Akt, and p-mTOR (**C**) were quantified as described in Materials and Methods. Data represent the mean ± SEM of three experiments. *** *p* < 0.001 vs. 2% FBS in pCMV-Entry-expressing cells; ^###^ *p* < 0.001 vs. 2% FBS in INS1E-hIAPP-overexpressing cells; ^&&^ *p* < 0.01, ^&&&^ *p* < 0.001, pCMV-Entry-expressing cells vs. INS1E-hIAPP-overexpressing cells.

**Figure 7:**
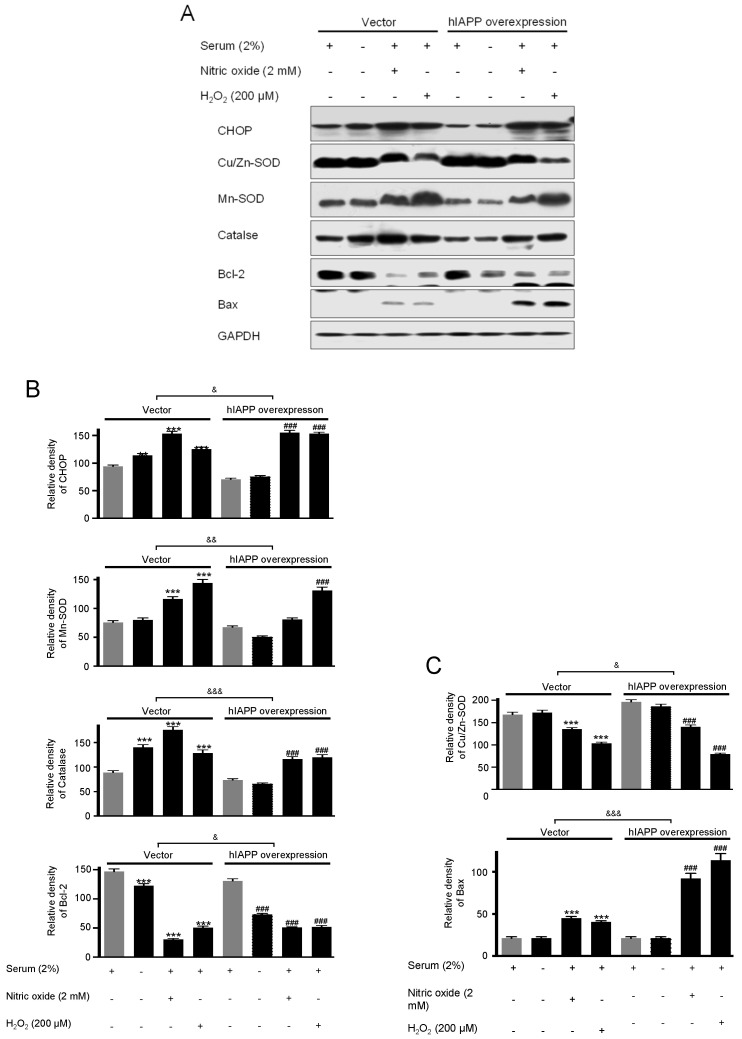
The expressions of CHOP, Cu/Zn-SOD, Mn-SOD, catalase, Bcl-2, and Bax proteins in pCMV-Entry-expressing cells and INS1E-hIAPP-overexpressing INS-1E cells under oxidative stress conditions. INS-1E cells were incubated in RPMI 1640 medium supplemented with/without 2% fetal bovine serum and/or nitric oxide (2 mM) or hydrogen peroxide (200 μM) for 24 h at 37 °C with 5% CO_2_. CHOP, Cu/Zn-SOD, Mn-SOD, catalase, Bcl-2, and Bax were then analyzed by Western blot (**A**). The relative amounts of CHOP, Mn-SOD, catalase, and Bcl-2 (**B**) and Cu/Zn -SOD, and Bax (**C**) were quantified as described in Materials and Methods. Data represent the mean ± SEM of three experiments. *** *p* < 0.001 vs. 2% FBS in pCMV-Entry-expressing cells; ^###^ *p* < 0.001 vs. 2% FBS in INS1E-hIAPP-overexpressing cells; ^&^ *p* < 0.05, ^&&^ *p* < 0.01, ^&&&^ *p* < 0.001, pCMV-Entry-expressing cells vs. INS1E-hIAPP-overexpressing cells.

**Figure 8:**
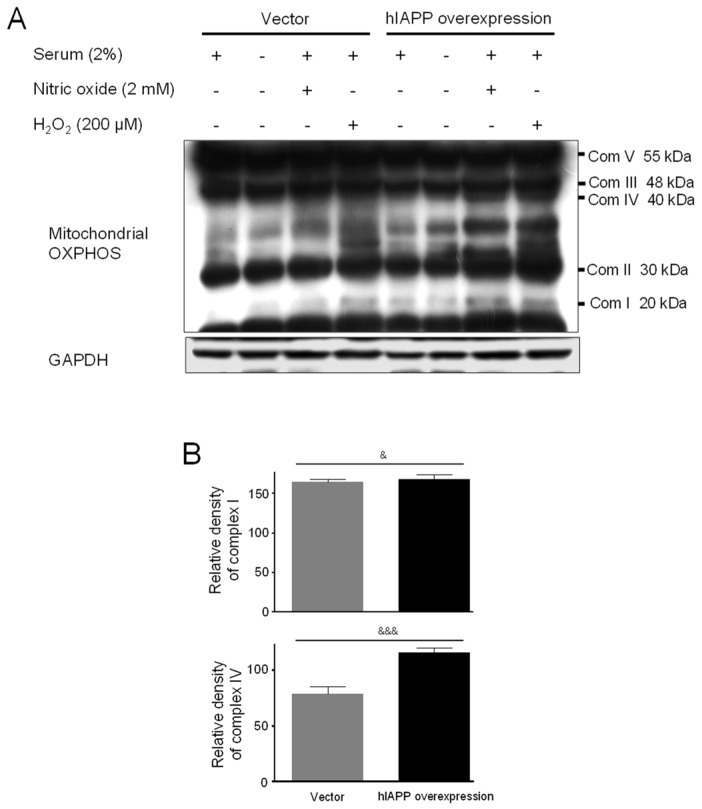
The effect of mitochondrial complex I and complex IV of OXPHOS in pCMV-Entry-expressing cells and INS1E-hIAPP-overexpressing INS-1E cells under oxidative stress conditions. INS-1E cells were incubated in RPMI 1640 medium supplemented with/without 2% fetal bovine serum and/or nitric oxide (2 mM) or hydrogen peroxide (200 μM) for 24 h at 37 °C with 5% CO_2_. Mitochondrial complex I and complex IV were then analyzed by Western blot (**A**). The relative amounts of mitochondrial complex I and complex IV (**B**) were quantified as described in Materials and Methods. Data represent the mean ± SEM of three experiments. ^&^ *p* < 0.05, ^&&&^ *p* < 0.001, pCMV-Entry-expressing cells vs. INS1E-hIAPP-overexpressing cells.

Second, we investigated hIAPP oligomerization/expression and signal pathways under oxidative stress in INS-1E cells. Oxidative stress is induced by serum deprivation, nitric oxide, or hydrogen peroxide treatments. Cell viability did not change in serum deprivation but significantly decreased in nitric oxide and hydrogen peroxide conditions (Figure 5). The pCMV-hIAPP-overexpressing cells pattern of amylin oligomerization/expression under oxidative stress was different from that of ER stress. Amylin oligomerization/expression under oxidative stress showed one band of amylin oligomers of less than 15 kDa in INS-1E cells. Under oxidative stress conditions (Figure 6), HIF1α, p-ERK, CHOP, Mn-SOD, catalase, and Bcl-2 were significantly reduced in the pCMV-hIAPP-overexpressing cells compared to the control. In contrast, p-mTOR, Cu/Zn-SOD, and Bax were significantly increased (Figure 6 and Figure 7). In mitochondrial OXPHOS, mitochondrial complex I and complex IV were significantly increased under oxidative stress conditions in pCMV-hIAPP-overexpressing INS-1E cells compared to the control (Figure 8).

### 2.3. Comparing Amylin Oligomerization/Expression and Cell Signaling under ER Stress and Oxidative Stress Conditions

Human amylin oligomerization/expression under ER stress showed three bands of amylin oligomers of less than 15 kDa size in pCMV-hIAPP-overexpressing cells ER stress and one band under oxidative stress (Figure 1, Figure 2, Figure 5 and Figure 6). HIF1α, p-ERK, and CHOP proteins were increased and decreased respectively under ER stress and oxidative stress, but Cu/Zn-SOD, Mn-SOD, catalase, Bcl-2, Bax, and mitochondrial complex I/IV proteins were reversely expressed (Figure 2, Figure 3, Figure 4, Figure 6, Figure 7 and Figure 8). These results suggest that ER stress has a more significant effect on intracellular amylin oligomerization than oxidative stress, and therefore has a significant contribution in inducing human pancreatic β-cell dysfunction and subsequent T2D and that ER stress and oxidative stress influence intracellular signal pathways differently, consequently leading to varied intracellular stress effects.

## 3. Discussion

Wong et al. [19] demonstrated that overexpression of hIAPP in INS-1 cells formed three oligomers between 4 and 8 kDa in size. Kim et al. [20] showed that hIAPP-transfected INS-1 cells expressed three oligomers of less than 17 kDa size under autophagy inhibition conditions, including inhibition by 3-methyladenine and bafilomycin. These results suggest that autophagy can eliminate amyloidogenic hIAPP and that autophagy deficiency contributes to the pathogenesis of human T2D. Burillo et al. [21] reported that overexpressing hIAPP in INS-1E cells accumulated one amylin protein of approximately 16 kDa under high glucose conditions, suggesting that this band corresponds to the aggregation of 16 kDa amylin protein as a tetramer. Chatterjee Bhowmick and Jeremic [22] revealed that human pancreatic islets induced three amylin oligomers of approximately 6 kDa in size under high glucose with/without proteasomal inhibitor, indicating a relation between hIAPP turnover and proteasome function in human islet β-cells. Chatterjee Bhowmick et al. [23] identified three amylin oligomers of approximately 6 kDa in size under high glucose (20 mM) media for 4 days and 0.5 μM thapsigargin (TG) for 24 h in human islets. They suggested that hIAPP biosynthesis is associated with ER-stressed human islet cells. ER stress may increase intracellular hIAPP levels by inhibiting the processing and/or secretion of amylin. Similarly, the present study results showed three bands of amylin oligomers of less than 15 kDa in size in INS-1E cells under ER stress and one oligomer under oxidative stress, suggesting that ER stress has a more significant effect on intracellular amylin oligomerization than oxidative stress. Additionally, this indicated that oligomerization differed depending on the cell-applied conditions and the amylin antibody used in the above studies. These results were obtained in vitro, but it is difficult to confirm the formation of amylin in vivo. A similar mechanism could facilitate the access of aggregated human amylin protein to the extra-pancreatic regions. This could be a plausible explanation for human amylin-induced human pancreatic β-cell dysfunction and subsequent T2D and Alzheimer’s disease [24,25].

The association between hIAPP-induced cytotoxicity and HIF1α activation is vital in the pancreatic β-cells. The activation of HIF1α and 6-phosphofructo-2-kinase/fructose 2,6 biphosphatase-3 (PFKFB3) metabolic pathway protects against hIAPP-induced β-cell apoptosis associated with T2D [26] and cytokine-induced β-cell death related to T1D [27]. Changes in the mitochondrial function and metabolism by hIAPP toxicity determine the role of β-cells in T2D [26,28,29]. hIAPP-induced β-cells toxicity is inhibited by protective metabolism induced by the HIF1α/PFKFB3 metabolic stress pathway, which slow cellular metabolism without blocking cellular dysfunction. That is, suppression of HIF1α activation enhances hIAPP-induced β-cytotoxicity. Wong et al. [19] showed that hIAPP aggregation in adenoviral hIAPP transduced INS-1E cells and human islet β-cells triggers mitochondrial apoptosis and blocks the release of cytochrome c or pro-apoptotic proteins Bax and Bak. The hIAPP aggregates were demonstrated to enhance the survival of β cells. Rivera et al. [30] reported that hIAPP aggregates impair the autophagy/lysosomal degradation pathway in pancreatic β cells, indicating that enhanced autophagy protects β-cells from hIAPP-induced apoptosis. However, overexpression of hIAPP in INS-1E cells induces MTORC1 activation and mitophagy inhibition [21,31]. These findings suggest that hIAPP aggregates can indirectly activate Bax/Bak, resulting in mitochondria dysfunction, oxidative stress, and ER stress. The fiber formation of hIAPP and amyloid aggregation causes extensive oxidative stress on pancreatic tissue. The binding of copper ions to hIAPP can produce ROS [32], resulting in a partial reduction of the β-cell mass. In practice, various cells components are transformed by ROS, acting as biomarkers for T2D and prediabetic stages [33,34]. In the present study, HIF1α in hIAPP-overexpressing cells significantly increased under ER stress, but HIF1α in INS-1E and hIAPP-overexpressing INS-1E cells significantly decreased under oxidative stress. Phospho-mTOR, Bcl-2, Bax, Mn-SOD, Cu/Zn-SOD, catalase, and mitochondrial complex I/IV related to mitochondrial function were investigated. Protein expressions of phospho-mTOR and mitochondrial complex I/IV between oxidative stress and ER stress were opposite, while the levels of Bcl-2, Bax, Mn-SOD, Cu/Zn-SOD, and catalase proteins between oxidative stress and ER stress were similar. ER stress and serum deprivation did not influence the INS -1E cell viability, but nitric oxide and hydrogen peroxide conditions induced cell death.

So far, various studies on the role of mitochondria in human diseases have been conducted with significant progress. Although mitochondria are central to energy metabolism, they play a critical role in apoptosis [35,36] and cell proliferation [37]. Mitochondria are also major organelles regulating calcium signaling as a secondary messenger [38,39]. The OXPHOS system produces mitochondrial ATP [40]. OXPHOS couples two sets of reactions, the phosphorylation of ADP and electron transfer through a chain of oxidoreductase reactions. Most eukaryotes consist of 5 enzyme complexes embedded in the mitochondrial inner membrane: complex I (NADH:ubiquinone oxidoreductase), complex II (Succinate:ubiquinone oxidoreductase), complex III (cytochrome c oxidoreductase or cytochrome bc1 complex), complex IV (cytochrome c oxidase), and complex V (ATP synthase). Complex I is the multimeric enzyme complex of the mitochondrial respiratory chain, which is responsible for electron transport and generating a proton gradient across the mitochondrial inner membrane to drive ATP production. Complex I dysfunction is the most common OXPHOS disorder in humans, and defects in the complex I assembly process are common [41,42]. In particular, complex I regulate reactive oxygen species (ROS), which are essential molecules in various signaling pathways, including apoptosis. The final enzyme complex IV reduces O_2_ to H_2_O in the electron transfer chain using the delivered electrons [43]. Through the pathway by complexes I, III, and IV, five protons per electron are pumped from the mitochondrial matrix into the intermembrane space, creating a membrane potential. Next, the transmembrane electrochemical potential promotes the conformational change of Complex V, resulting in the generation of ATP [44].

One component of ER stress-mediated apoptosis pathway is C/EBP homologous protein (CHOP) which is associated with cell death by growth arrest and DNA damage. Thus, CHOP as an ER stress-mediated apoptotic factor plays a significant role in the pathogenesis of diabetes, ischemia, and neurodegenerative diseases [45]. CHOP-deleted mice showed reduced apoptosis in response to ER stress [46,47,48]. On the other hand, CHOP overexpression reduces Bcl-2 protein and translocates Bax protein from the cytosol to the mitochondria [49,50]. Therefore, eventually, CHOP-mediated death signals affect the mitochondria as an essential factor in the path of cell death. The high expression levels of hIAPP in INS-1 cells and human IAPP transgenic rats induce ER stress-mediated β-cell apoptosis and increase CHOP expression [51,52,53], suggesting that oligomerization by hIAPP can be toxic and cause ER stress-induced apoptosis. ER stress plays a causative role in beta-cell dysfunction in overexpressing hIAPP [54]. hAIPP transgenic and non-transgenic mouse islets induce oxidative stress, and amyloid formation mediates β-cell apoptosis [55,56]. In the present study, ER stress and oxidative stress influenced the CHOP protein levels in hIAPP-overexpressing cells with the change of Bcl-2 and Bax protein levels. 

So far, very few studies have discussed ERK and Akt’s activation under ER stress and oxidative stress conditions by hIAPP overexpression. In this study, the ER stress caused by hIAPP overexpression did not affect cell survival and seemed to involve the Akt/mTOR pathway. On the other hand, oxidative stress conditions reduced cell survival without the effect of hIAPP overexpression, which was associated with phospho-ERK reduction. hIAPP overexpression induces β-cell apoptosis by regulating Fak and Akt phosphorylation in the β-cells [57]. Notably, Cyclin-dependent kinase 5 (CDK5) is especially required for neuronal survival by activating ERK and PI3K/Akt pathways [58,59]. The loss of CDK5 in both INS 832/13 cells and primary β-cells resulted in an attenuation of PI3K/Akt survival pathway and increased β-cell apoptosis.

## 4. Materials and Methods

### 4.1. Cell Culture and Induction of ER Stress and Oxidative Stress

The INS-1E cells were cultured in RPMI 1640 medium (Invitrogen, Carlsbad, CA, USA) containing 11 mM glucose supplemented with 10 mM HEPES (pH 7.3), 10% heat-inactivated fetal bovine serum (FBS; Invitrogen), 50 μM β-mercaptoethanol, 1 mM sodium pyruvate, 50 μg/mL penicillin, and 100 μg/mL streptomycin at 37 °C with 5% CO_2_ in a humidified incubator. ER stresses are induced by thapsigargin (1 μM; Calbiochem, San Diego, MO, USA) for 6 h or tunicamycin (2 μg/mL; Calbiochem) for 16 h treatments with/without 4-phenylbutyric acid (4-PBA, 20 μM; Santa Cruz Biotechnology, Santa Cruz, CA, USA). Oxidative stresses are induced by serum deprivation, nitric oxide (2 mM; Santa Cruz Biotechnology), or hydrogen peroxide (200 μM; Santa Cruz Biotechnology) for 24 h treatments.

### 4.2. INS-1E Stable Cell Line by Overexpressing hIAPP

CMV6-Entry (control) and pCMV6-hIAPP vectors were purchased from OriGene Technologies Inc. (Rockville, MD, USA). These vectors were transfected individually into INS-1E cells Opti-MEM^®^ (Gibco BRL, CA, USA). Stably transfected cells were selected in 200 µg/mL G418 containing growth media and thus pCMV-hIAPP-overexpressing cells and pCMV-Entry-expressing cells (control).

### 4.3. Cell Viability Assay

According to the manufacturer’s protocol, cell viability was determined using a Cell Counting Kit-8 (Dojindo Molecular Technologies, Inc.). Cells were plated in 96-well plates (Corning, Inc., Corning, NY, USA) at 5 × 10^3^/well density. The cells were treated with the 10 μL kit solution, incubated for 30 min, and their absorbance was measured at 450 nm. Percent viability was calculated as follow: Cell Viability (%) = [(Total absorbance-Background absorbance)/Control absorbance] × 100.

### 4.4. Western Blot Analysis 

Cell extracts were lysed in 20 mM Tris-HCl buffer (pH 7.4) containing 0.1 mM PMSF, and 1:100 protease inhibitor cocktail (Sigma, St Louis, MI, USA) at 4 °C. Protein concentrations were determined using the BCA assay (Sigma). Proteins (40 µg) resolved by 10% Gradi-Gel II gradient PAGE (ELPIS-Biotech, Daejeon, Korea). After gel electrophoresis, the protein was transferred to a polyvinylidene difluoride (PVDF) membrane. The membrane was incubated with antibodies. The amylin antibody (catalog no. LS-C352341; 1:1000) was provided from LifeSpan BioSciences, Phospho-ERK (catalog no. sc-7380; 1:500), ERK (catalog no. sc-93; 1:500), Cu/Zn-SOD (catalog no. sc-271014; 1:500), Mn-SOD (catalog no. sc-137254; 1:500), C/EBP-homologous protein (CHOP; catalog no. sc-575; 1:500) Bcl-2 (catalog no. sc-7382; 1:500), Bax (catalog no. sc-7480; 1:500), and GAPDH (catalog no. sc-25778; 1:500) obtained from Santa Cruz Biotechnology (Santa Cruz). Hypoxia-inducible factor (HIF)-1α (catalog no. 14179, 1:1000) p-Akt (catalog no. 4051; 1:1000), p-mTOR (catalog no. 2971; 1:1000), and catalase (catalog no. 12980; 1:1000) were provided by Cell Signaling Technology, Inc. (Danvers, MA, USA) Subsequently, the membranes were incubated with anti-mouse IgG (catalog no. 7076; 1:1000; Cell Signaling Technology, Inc.) or anti-rabbit IgG secondary antibodies conjugated to HRP (catalog no. 7074; 1:1000; Cell Signaling Technology, Inc.) for 1 h in room temperature. Protein bands were detected with chemiluminescent substrate (Thermo Fisher Scientific, Inc., Waltham, MA, USA) and then measured using ImageJ software (version 1.37; National Institute of Health).

### 4.5. Statistical Analysis 

Significant analyses were determined by using ANOVA followed by Tukey’s test for multiple comparisons with the Prism Graph Pad v4.0 (Graph Pad Software, San Diego, CA, USA). Values are expressed as means ± SEM of at least three separate experiments. *p* values < 0.05 were considered statistically significant.

## 5. Conclusions

We tried to compare human amylin oligomerization/expression and signal transduction under ER stress and oxidative stress. Human amylin undergoes protein oligomerization under ER stress in hIAPP-overexpressing INS-1E cells. ER stress has a more significant effect on intracellular amylin oligomerization than oxidative stress. ER stress and oxidative stress influence intracellular signal pathways differently, consequently leading to varied effects of intracellular stress. Therefore, human amylin overexpression with ER stress significantly contributes to inducing human pancreatic β-cell dysfunction and subsequent T2D.

## Data Availability

The data presented in this study are available on request from the corresponding author.
